# Assessing the external validity of machine learning-based detection of glaucoma

**DOI:** 10.1038/s41598-023-27783-1

**Published:** 2023-01-11

**Authors:** Chi Li, Jacqueline Chua, Florian Schwarzhans, Rahat Husain, Michaël J. A. Girard, Shivani Majithia, Yih-Chung Tham, Ching-Yu Cheng, Tin Aung, Georg Fischer, Clemens Vass, Inna Bujor, Chee Keong Kwoh, Alina Popa-Cherecheanu, Leopold Schmetterer, Damon Wong

**Affiliations:** 1 Singapore Eye Research Institute, Singapore National Eye Centre, 20 College Road, The Academia, Level 6, Discovery Tower, Singapore, 169856 Singapore; 2 Ophthalmology & Visual Sciences Academic Clinical Program, Duke-NUS Medical School, Singapore, Singapore; 3 SERI-NTU Advanced Ocular Engineering (STANCE), Singapore, Singapore; 4 School of Computer Science and Engineering, Nanyang Technological University, Singapore, Singapore; 5 Center for Medical Statistics Informatics and Intelligent Systems, Section for Medical Information Management, Medical University Vienna, Vienna, Austria; 6 Department of Clinical Pharmacology, Medical University Vienna, Vienna, Austria; 7 Institute of Molecular and Clinical Ophthalmology, Basel, Switzerland; 8 Department of Ophthalmology, Yong Loo Lin School of Medicine, National University of Singapore, Singapore, Singapore; 9 Department of Ophthalmology and Optometry, Medical University Vienna, Vienna, Austria; 10 Carol Davila University of Medicine and Pharmacy, Bucharest, Romania; 11 Department of Ophthalmology, Emergency University Hospital, Bucharest, Romania; 12 School of Chemical and Biomedical Engineering, Nanyang Technological University, Singapore, Singapore; 13 Center for Medical Physics and Biomedical Engineering, Medical University Vienna, Vienna, Austria

**Keywords:** Optic nerve diseases, Pathology

## Abstract

Studies using machine learning (ML) approaches have reported high diagnostic accuracies for glaucoma detection. However, none assessed model performance across ethnicities. The aim of the study is to externally validate ML models for glaucoma detection from optical coherence tomography (OCT) data. We performed a prospective, cross-sectional study, where 514 Asians (257 glaucoma/257 controls) were enrolled to construct ML models for glaucoma detection, which was then tested on 356 Asians (183 glaucoma/173 controls) and 138 Caucasians (57 glaucoma/81 controls). We used the retinal nerve fibre layer (RNFL) thickness values produced by the compensation model, which is a multiple regression model fitted on healthy subjects that corrects the RNFL profile for anatomical factors and the original OCT data (measured) to build two classifiers, respectively. Both the ML models (area under the receiver operating [AUC] = 0.96 and accuracy = 92%) outperformed the measured data (AUC = 0.93; P < 0.001) for glaucoma detection in the Asian dataset. However, in the Caucasian dataset, the ML model trained with compensated data (AUC = 0.93 and accuracy = 84%) outperformed the ML model trained with original data (AUC = 0.83 and accuracy = 79%; P < 0.001) and measured data (AUC = 0.82; P < 0.001) for glaucoma detection. The performance with the ML model trained on measured data showed poor reproducibility across different datasets, whereas the performance of the compensated data was maintained. Care must be taken when ML models are applied to patient cohorts of different ethnicities.

## Introduction

Glaucoma is characterized by progressive loss of retinal ganglion cells, leading to visual impairment^1^. Given that visual loss from glaucoma is irreversible, the disease must be detected at an early stage before significant visual field (VF) loss has been established so that the risk of vision-related morbidity can be minimized^2^. While the disease is diagnosed based on funduscopic examination and VF testing, optical coherence tomography (OCT) is an efficient approach to support clinicians since it provides quantitative, reproducible measurements which are less patient-dependent^3^.

Recently, machine learning (ML) models have been applied to OCT structural data to assist with glaucoma diagnosis^4^. ML classifiers are computerized systems trained with the capacity to identify the relationship between multiple features and a disease diagnosis. While most have reported high diagnostic accuracies (AUC = 0.88–0.98) for glaucoma detection^5–9^, none assessed the models with independently sampled data from a different ethnicity group (external test), which limits the generalizability of the models across ethnicities^10^.

Anatomic factors such as retinal vessel tree configuration, fovea-disc angle, or optic disc size vary across ethnicities^11–14^ and can affect peripapillary retinal nerve fibre layer (RNFL) thickness^15–21^. We have previously developed a compensation model, which is a multiple regression model fitted on healthy subjects that corrects the RNFL profile for factors like ethnicity, disc parameters and age^22^, showing better glaucoma discrimination capability than original OCT data (measured RNFL thickness)^23^. It is likely that ML models trained on compensated data may be more applicable across ethnicities since the classifier would have access to “distilled” information and would not be biased by the anatomic factors.

The objective of this paper was to validate ML models for glaucoma detection from OCT data (Cirrus, Carl Zeiss Meditec, Dublin, CA). We used RNFL thickness values produced by the compensation model and the original Cirrus-generated data (measured) to build two classifiers, respectively.

## Materials and methods

### Study population

Glaucoma patients and controls were enrolled from the Singapore Eye Institute in Singapore and the Department of Ophthalmology, Emergency University Hospital, Bucharest, Romania, respectively. The Asian study was approved by the human ethics boards of Singapore Eye Research Institute and the Caucasian study by the Emergency University Hospital Bucharest Ethics Committee. Both studies were conducted according to the Declaration of Helsinki and written informed consent was obtained from all participants.

For the Asian study, glaucoma cases^24^ were enrolled in the Singapore Imaging Eye Network (SIENA) study from July 2018 to July 2019 and controls^25^) from the Singapore Epidemiology of Eye Disease (SEED) program. Briefly, SIENA is a clinical cross-sectional study investigating the effects of vascular abnormalities in participants with primary open-angle glaucoma (POAG) aged 21 years and older, and SEED study is a population-based study comprised of Chinese, Malays and Indians aged 40–80 years living in Singapore^26^. Of the total 569 Asians with glaucoma, 129 had missing variables or poor-quality scans, leaving a final sample of 440 glaucoma patients (n = 513 eyes), of which 370 were Chinese, 42 Malays and 27 Indians and 1 individual of mixed Asian origin. Of the 3988 Asians with normal eyes, 1289 had missing variables or poor-quality scans, leaving a sample of 2699 controls^25^. For the present analysis, we applied a frequency matching technique based on age- (by 5-year age groups), gender- and race-matching^25^, where one control was randomly selected for each glaucoma participant. Therefore, 430 controls (n = 513 eyes) comprising of 348 Chinese, 48 Malays and 34 Indians participants were included for the analysis.

For the Caucasian study, glaucoma cases and controls were enrolled from the Department of Ophthalmology, Emergency University Hospital, Bucharest, Romania from August 2020 to March 2021. From a total of 78 glaucoma patients and 105 controls, 45 with missing or poor-quality scans were excluded, leaving 57 glaucoma patients (n = 87 eyes) and 81 controls (n = 145 eyes) for the final analysis (Supplementary Fig. S1).

Study methodologies were identical for Asians and Caucasians unless otherwise stated. Participants underwent an ocular examination which included measurement of visual acuity using a logarithm of the minimum angle of resolution chart (LogMAR chart, The Lighthouse, NY), auto-refraction-keratometry (Asian: Canon RK-5 Autorefractor Keratometer; Canon Inc., Tokyo, Japan; Caucasian: CHAROPS CRK-7000; Huvitz Co., Ltd, South Korea)^27^, axial length measurement (Asian: IOL Master V3.01, Carl Zeiss Meditec AG, Jena, Germany; Caucasian: IOL Master V5, Carl Zeiss Meditec AG, Jena, Germany), intraocular pressure measurement using Goldmann applanation tonometry, and OCT imaging (see later section). Only glaucoma participants underwent Humphrey Swedish interactive thresholding model SITA standard or SITA fast 24–2 perimetry (Asian: Humphrey VF analyser II; Carl Zeiss Meditec, USA; Caucasian: Zeiss Humphrey Field Analyzer 3, Model 860 Carl Zeiss Meditec, USA). Spherical equivalent (SE) was calculated as the spherical value plus half of the negative cylinder value.

Glaucoma patients were defined during an ophthalmic examination by board certified ophthalmologists following the criteria: presence of glaucomatous optic neuropathy (defined as loss of neuroretinal rim with a vertical cup: disc ratio of > 0.7 or an inter-eye asymmetry of > 0.2 and/or notching attributable to glaucoma) with compatible and reproducible VF in standard automated perimetry, and open angles on gonioscopy. Patients with secondary glaucoma or history of retinal operation or laser treatments were excluded from this study. Glaucoma severity was defined on perimetry findings as mild glaucoma (mean deviation (MD) ≥ -6 dB, moderate glaucoma (-6.01 to -12.00 dB), and severe glaucoma (MD <−12 dB)^28,29^. Controls were defined as individuals free from clinically relevant eye conditions and were confirmed to have no sign of glaucoma or other major eye diseases except for mild cataract by an ophthalmic examination. These controls at time of recruitment had intraocular pressure < 21 mmHg with open angles, healthy optic nerves, and no family history of glaucoma.

### OCT Imaging

Participants underwent Cirrus (Carl Zeiss Meditec, Dublin, CA) spectral domain-OCT optic disc and macular cube (200 × 200) imaging after pharmacological dilation^30^. Trained graders, masked to the participant’s characteristics reviewed the quality of OCTs. Only eyes with good quality images (signal strength greater than or equal to 6), no excessive movement artifacts, consistent signal intensity across the scan, circular scan centred at the optic disc, and no segmentation artifact were included in the analysis. Both eyes were included in the study if they met the eligibility criteria. Supplementary Fig. S2 shows two representative OCT images of normal and glaucoma patients.

### Extractions of measured RNFL thickness and other ocular anatomical features

Peripapillary RNFL thickness measurements were automatically obtained from a 3.46 mm diameter ring around the optic disc using the Cirrus Review Software (software version 11.0.0.29946). The global RNFL thickness was calculated from 12 sector-wise values and used for subsequent comparative analysis.

Optic disc and macular OCT images were processed with MATLAB (MathWorks Inc., R2018b, Natick, MA) to extract anatomical features such as the optic disc, fovea, and the retinal vessel tree from the OCT volumes^22^. Optic disc variables including its area, orientation (angle between the horizontal axis and the major axis of the optic disc), and ratio (between major and minor axes) were extracted from the OCT. The optic disc ratio describes how elliptical the optic disc is, where a value closer to 1.0 indicates a more circular optic disc. From the registered optic disc and macula images, the fovea distance (the distance between optic disc and fovea centers) and fovea angle (the angle between a line connecting fovea and optic disc centers and a horizontal line passing through the optic disc center) were calculated.

### Compensation model

We previously developed a compensation model, which is a multiple regression model fitted on healthy subjects that corrects the RNFL profile for eight factors, namely age, refractive error, optic disc (ratio, orientation, and area), fovea (distance and angle), and retinal vessel density^23^. A compensation model was developed separately for each ethnic group (Chinese, Malay and Indian). It was trained on 1619 healthy Asian patients (n = 2389 eyes).

### Machine learning models

We used the Asian dataset and built two classifiers, one using the RNFL thickness values produced by the compensation model and the original Cirrus-generated data (measured) (Supplementary Fig. S1). We used the 12 sectors of RNFL thickness values to build the ML models. The Asian dataset was randomly divided using a 6:4 ratio for the training and test datasets based on the age, gender, and race frequency matching, where the training set consists of 257 glaucoma patients (n = 307 eyes) and 257 controls (n = 307 eyes), and the internal test set consists of 183 glaucoma patients (n = 206 eyes) and 173 controls (n = 206 eyes; Supplementary Fig. S1). We compared the performance of the ML models on our internal Asian dataset of 356 participants (412 eyes) and the external Caucasian dataset comprising of 138 participants (232 eyes).

We combined four different ML models, logistic regression (LR)^31^, support vector machines (SVM)^31^, random forests (RF)^32^, and gradient boosting (GB)^33^, using soft voting ensembling (SVE). LR analysis involves fitting a logit function for class prediction, while a SVM classifier works by determining the hyperplane for maximal class separation. In contrast, RF classifiers are a collection of decision trees, each constructed by randomly sampling from the training data with replacement in a process known as bagging. GB classifiers are decision tree models in which a sequence of decision trees is generated, with each subsequent tree correcting for errors made by prior trees. While each of these ML models may be used for glaucoma detection, SVE balances out the potential weaknesses of each model by combining their inputs^34^. Class probabilities obtained from each classifier were averaged and the class with the highest averaged probability was used as the output of the SVE.

Grid search fivefold cross-validation is used for finetuning of the hyperparameters for each specific classifier. Apart from some pre-determined parameters, like penalty and kernel, the remaining parameters are estimated with GridSearchCV in sklearn.model_selection. GridSearchCV is a way of iterating through the possible values for target parameter and testing the accuracy through cross validation. The dataset used for model training was first separated into five groups. First, the cross validation iterates through the folds and uses one of the five folds as the validation dataset at each iteration while all remaining folds are used as training dataset. This process is repeated until every fold has been used as a validation dataset for every combination of potential value of the parameters.

### Statistical analyses

The characteristics between train and test datasets were compared using independent t-test and chi-square for continuous and categorical variables, respectively. Independent *t* test and chi-squared test were conducted in R version 4.0.4 (The R Foundation for Statistical Computing) using functions t.test and chisq.test. The area under the receiver operating characteristics curve (AUC) for glaucoma detection were calculated. To account for the nested structure of dataset, eyes of same patient were clustered in ROC analyses. 1000 bootstrap replicates from the internal and external datasets were drawn to generate the 95% bootstrap percentile intervals. Sensitivity at 95% specificity was also generated. DeLong’s test was conducted in R version 4.0.4 (The R Foundation for Statistical Computing), using function roc.test in pROC packages. DeLong’s test evaluated the difference between AUCs^35^. Accuracy was defined as the ratio between the number of properly classified samples over the overall number of samples^36^. F1 score was calculated as the harmonic mean of the precision and recall^36^. Spearman’s rank correlation coefficients (*r*_*s*_) were used to evaluate the relationship between VF’s mean deviation (VFMD) and raw values of structural damage either using measured RNFL or probability score predicted from ML models. A locally weighted scatterplot smoothing (LOWESS) curve was used to visualize the relationship between these variables. In this paper, statistical significance threshold was set at 0.05 as proposed by Fischer^37^. The exact P value for t-test, chi-squared test and DeLong’s test was also provided in Tables 1 and 2. Data were analyzed with R version 4.0.4 (The R Foundation for Statistical Computing).

**Table 1:** Characteristics of the training and testing datasets.

	Training Asian dataset	Testing Asian dataset	Testing Caucasian dataset	*P value	†P value
Number of patients	514	356	138	–	–
Age, years	63 ± 9	63 ± 9	49 ± 17	0.515	** < 0.001**
Gender, male	333 (65)	233 (65)	48 (35)	0.840	** < 0.001**
Race, Chinese	420 (82)	298 (84)	–	0.888	
Number of eyes	614	412	232	–	–
Normal	307	206	145	–	–
Glaucoma	307	206	87	0.959	** < 0.001**
Mild	188 (61)	120 (58)	65 (75)	–	–
Moderate	69 (23)	51 (25)	14 (16)	–	–
Severe	50 (16)	35 (17)	8 (9)	–	–
Number of normal patients, eyes	257, 307	173, 206	81, 145		
Fovea distance, mm	150.91 ± 8.66	149.23 ± 8.38	148.27 ± 8.21	**0.029**	** < 0.001**
Fovea angle, degrees	− 7.81 ± 3.62	− 7.71 ± 3.36	− 6.73 ± 2.98	0.750	** < 0.001**
Optic disc area, mm^2^	1.86 ± 0.37	1.89 ± 0.37	1.83 ± 0.36	0.480	0.383
Optic disc ratio	1.13 ± 0.07	1.14 ± 0.09	1.10 ± 0.05	0.239	** < 0.001**
Optic disc orientation, degrees	95.13 ± 32.01	97.11 ± 30.12	103.03 ± 28.82	0.479	**0.009**
Retinal vessel density, global	3.52 ± 0.53	3.49 ± 0.52	3.76 ± 0.54	0.466	** < 0.001**
Spherical equivalent refractive error, dioptres	− 0.44 ± 2.17	− 0.26 ± 1.96	− 0.14 ± 2.01	0.342	0.153
Global RNFL thickness, µm	94.14 ± 9.86	95.01 ± 10.32	95.06 ± 8.41	0.342	0.306
Number of glaucoma patients, eyes	257, 307	183, 206	57, 87		
Fovea distance, mm	149.67 ± 9.52	148.48 ± 9.84	148.99 ± 8.61	0.174	0.528
Fovea angle, degrees	− 7.90 ± 3.34	− 7.97 ± 4.01	− 7.23 ± 3.62	0.836	0.125
Optic disc area, mm^2^	2.01 ± 0.55	1.98 ± 0.52	2.00 ± 0.34	0.509	0.974
Optic disc ratio	1.15 ± 0.10	1.15 ± 0.10	1.08 ± 0.05	0.776	** < 0.001**
Optic disc orientation, degrees	90.04 ± 34.80	93.10 ± 33.26	109.66 ± 30.27	0.318	** < 0.001**
Retinal vessel density, global	3.38 ± 0.57	3.34 ± 0.55	3.59 ± 0.50	0.372	** < 0.001**
Spherical equivalent refractive error, dioptres	− 1.48 ± 3.13	− 1.70 ± 3.11	+ 0.37 ± 1.07	0.417	** < 0.001**
Global RNFL thickness, µm	72.43 ± 11.86	71.34 ± 12.03	80.54 ± 13.10	0.312	** < 0.001**

Data are number, mean ± standard deviation, or number (%). For n (%), the denominator is number of patients or number of eyes (by eye), as applicable.

P value was obtained with independent t-tests for continuous variables and with chi-square tests for categorical variables and values in bold indicate statistical significance.

* Denotes comparison between Asian training and Asian test dataset whereas † between Asian training and Caucasian test dataset.

**Table 2:** Comparison of glaucoma detection between measured retinal nerve fibre layer (RNFL), compensated RNFL, and machine learning approaches.

	Area under the receiver operating characteristic curve	Sensitivity at 95%	P value		
Testing Asian dataset					
1. Measured RNFL	0.93 (0.91–0.96)	0.76	Reference		
2. Compensated RNFL	0.93 (0.91–0.95)	0.73	0.531	Reference	** < 0.001**
3. Machine learning (with measured RNFL)	0.96 (0.95–0.98)	0.85	** < 0.001**	** < 0.001**	0.113
4. Machine learning (with compensated RNFL)	0.96 (0.94–0.98)	0.84	** < 0.001**	** < 0.001**	Reference
Testing Caucasian dataset					
1. Measured RNFL	0.82 (0.74–0.89)	0.49	Reference		
2. Compensated RNFL	0.93 (0.88–0.97)	0.67	** < 0.001**	Reference	0.641
3. Machine learning (with measured RNFL)	0.83 (0.75–0.90)	0.52	0.535	**0.002**	** < 0.001**
4. Machine learning (with compensated RNFL)	0.93 (0.89–0.97)	0.64	** < 0.001**	0.668	Reference

Data in parentheses are 95% confidence intervals.

Results for sensitivity are expressed as percentages.

P value was obtained with DeLong et al. (1988) for paired receiver operating characteristic curves and values in bold indicate statistical significance.

## Results

There mainly were no significant differences between the training dataset and the Asian test dataset (P ≥ 0.174), except for fovea distance (P = 0.029, Table 1). However, there were significant differences between the training dataset and the Caucasian test dataset, with participants in the external test dataset being younger (mean ± standard deviation (SD) of 49 ± 17 years), having a larger ratio of females, and more mild and moderate glaucoma eyes (P < 0.001, Table 1). The ocular characteristics of normal participants were also significantly different between the training dataset and the Caucasian test dataset, fewer Caucasians having significantly shorter fovea distances, smaller foveal angles, less elliptical optic discs (ratio closer to 1.0), and thicker retinal vessel densities (P ≤ 0.009, Table 1). For the glaucoma dataset, Caucasians had significantly less elliptical optic discs, higher optic disc orientations, thicker retinal vessel densities, were more hyperopic, and had larger RNFL thicknesses (P ≤ 0.001, Table 1).

**Figure 1:**
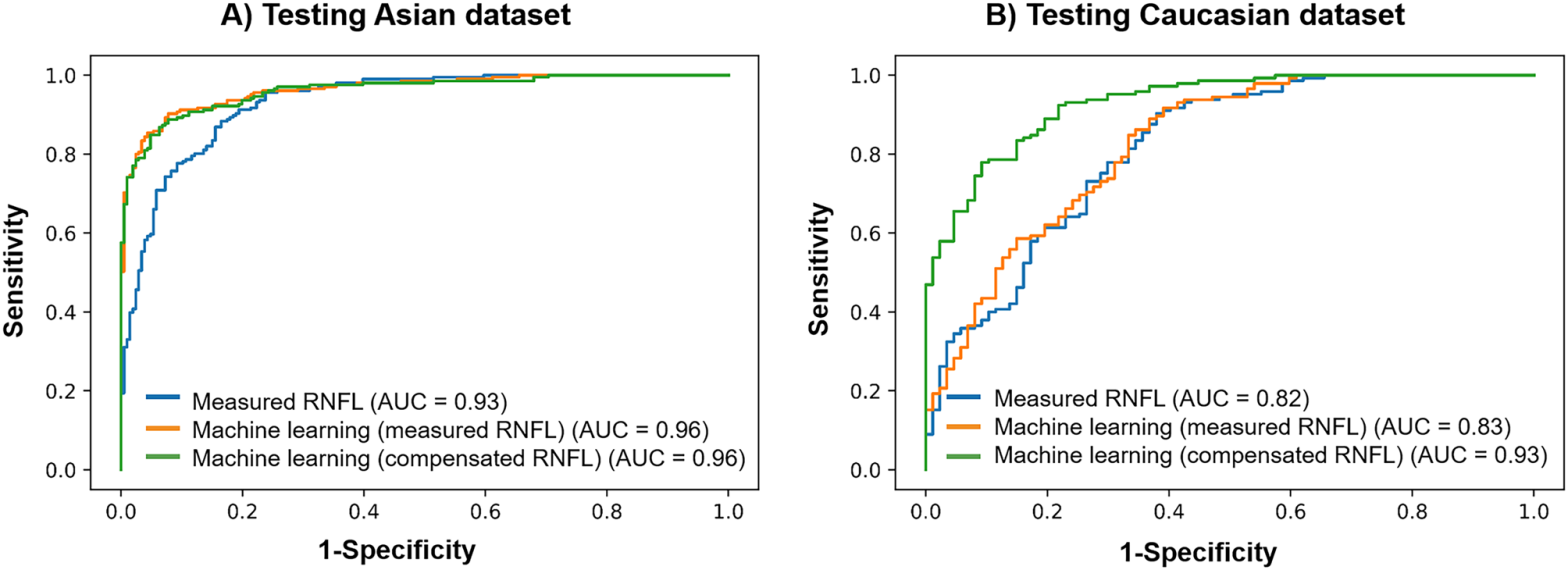
Receiver operating characteristic curves comparing glaucoma detection on the (**A**) Testing Asian dataset and (**B**) Testing Caucasian dataset using the measured retinal nerve fiber layer (RNFL), machine learning models using either measured RNFL thickness or compensated RNFL thickness. Values in brackets indicate the area under the curves (AUC) for the corresponding approaches.

The values for AUC and sensitivity at 95% specificity for glaucoma detection using the measured RNFL, compensated RNFL, and ML models trained either with measured RNFL or compensated RNFL (Table 2). In the Asian test dataset, both the ML models (AUC = 0.96) outperformed the measured RNFL thickness (AUC = 0.93; P < 0.001) and compensated RNFL thickness (AUC = 0.93; P < 0.001) for glaucoma detection. However, in the Caucasian dataset, the ML model trained with compensated data (AUC = 0.93) outperformed the ML model trained with measured RNFL (AUC = 0.83; P < 0.001) and measured RNFL thickness (AUC = 0.82; P < 0.001). There was no difference in the AUCs between the ML model trained with compensated data and compensated RNFL thickness (AUC = 0.89; P = 0.641). At a specificity level of 95%, the sensitivity for detecting glaucoma was highest using ML models (84–85%) and lowest using non-ML approaches (73–76%) in the Asian dataset (Table 2 and Fig. 1). In the Caucasian dataset, the sensitivity for detecting glaucoma was highest using compensated RNFL approaches (64–67%) and lowest using original RNFL (49–52%; Table 2 and Fig. 1). We also included the result of every classifier for every dataset (Supplementary Table S2) and the ML confusion matrix for testing of every dataset (Supplementary Table S3). The findings of Tables S2 and S3 support the overall finding that the use of compensated OCT data in ML achieves better results than measured data.

We next performed a separate sub-analysis on a balanced Caucasian test dataset using 87 normal and 87 glaucoma patients. Our additional findings were consistent with earlier analysis where the use of compensated data for machine learning (AUC = 0.92) is significantly better than measured data (AUC = 0.84; P < 0.01; Supplementary Table S1).

**Figure 2:**
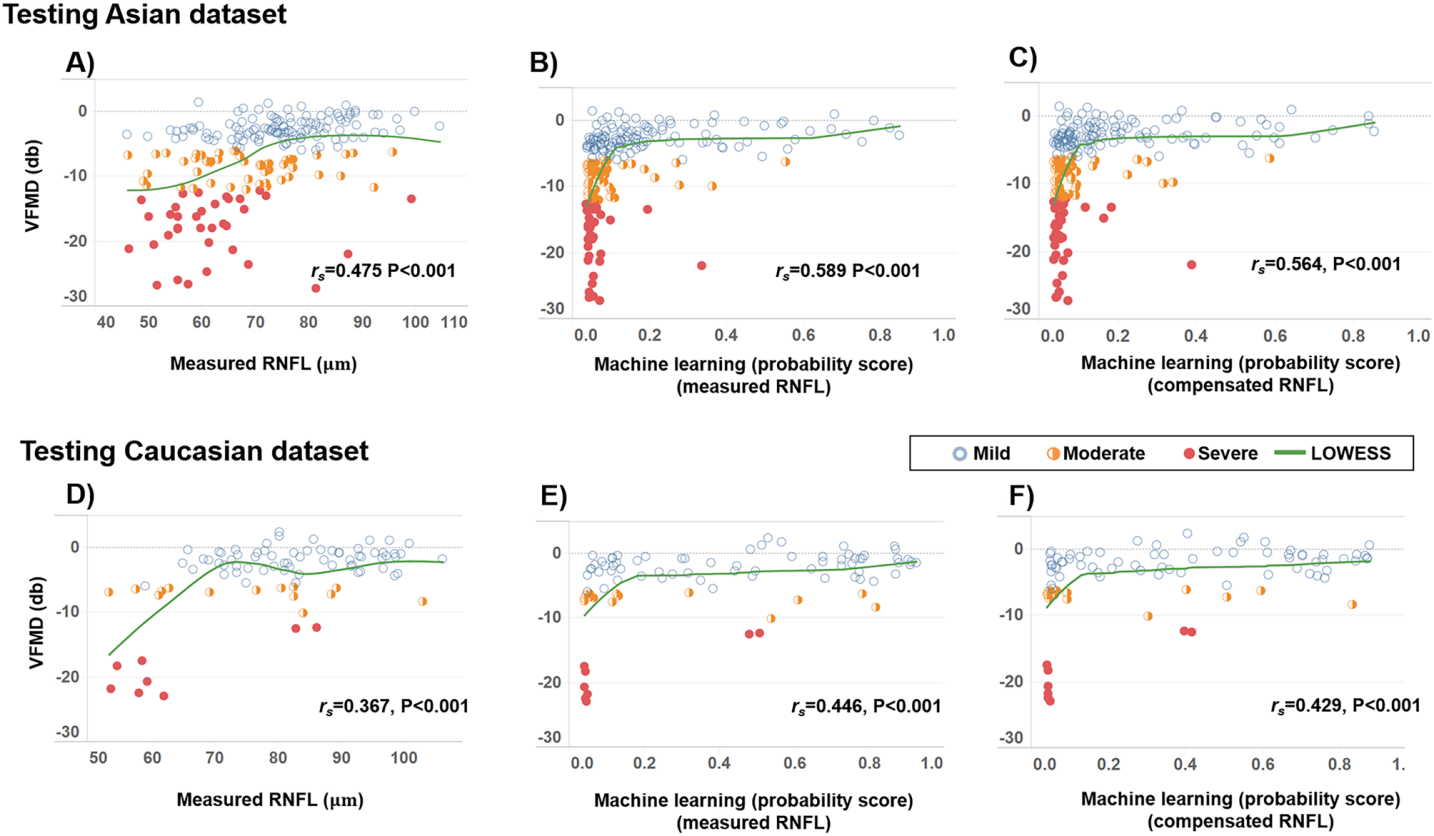
Scatter plots showing the associations between the visual field mean deviation (VFMD) and measured retinal nerve fiber layer (RNFL) thickness for (**A**) Asian and (**D**) Caucasian test datasets. (**B**) and (**E**) show the associations between VFMD, and the probability score generated by a machine learning (ML) model using measured RNFL thickness, where a lower score indicates greater severity of glaucoma. (**C**) and (**F**) show the associations between VFMD, and the probability score generated by a ML model using compensated RNFL thickness, where a higher score indicates greater severity of glaucoma. Spearman’s correlation coefficient ρ and LOWESS curves are indicated for the corresponding variables in each plot.

We evaluated the relationship between the VFMD and structural damage with the measured RNFL and ML models. Figure 2 shows the LOWESS plots, suggesting a monotonic relationship between VFMD and all approaches for both the internal test (+ 1.4 to − 27.3 dB) and external test (+ 2.5 to − 23.0 dB) datasets. This association with the VFMD was statistically significant for all approaches (P < 0.001). It was the strongest with the ML model trained with the measured RNFL in the Asian dataset (*r*_*s*_ = 0.589) and Caucasian dataset (*r*_*s*_ = 0.446).

**Figure 3:**
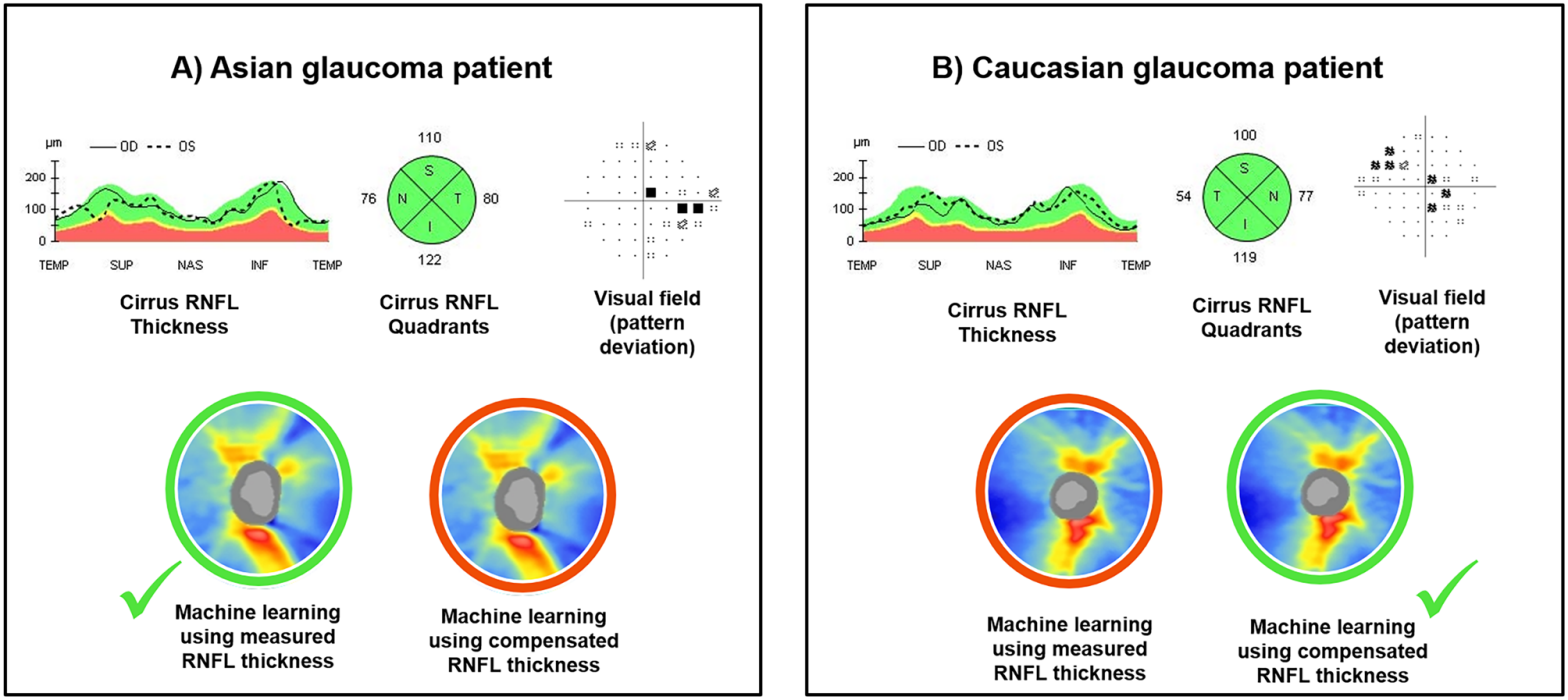
Representative glaucoma cases showing the diagnostic performances of the measured retinal nerve fiber layer (RNFL), machine learning model using measured RNFL thickness, and machine learning model using compensated RNFL thickness. Case (**A)** is an Asian glaucoma patient, while Case (**B**) is a Caucasian glaucoma patient. The Cirrus RNFL thickness maps indicate a reduction in the RNFL thickness in the superior and inferior regions in Case (**A**), while it shows a reduction in the superior regions in Case (**B**). However, the Cirrus quadrant analyses show a normal RNFL probability color code in all quadrants in both cases. The output prediction of both compensation and machine learning approaches are color-coded, where the red circle indicates wrong prediction, and the green circle indicates correct prediction. The machine learning model measured RNFL thickness correctly predicted Case (**A**) with glaucoma while the machine learning model using compensated RNFL thickness correctly predicted Case (**B**) with glaucoma.

We reviewed two glaucoma cases in which there are differences in the results between the Cirrus-generated RNFL analysis and the ML models (Fig. 3). Case A is an Asian glaucoma patient presenting a normal RNFL probability color code in all quadrants with the Cirrus normative database and ML with compensated RNFL. Only the ML model using measured RNFL correctly predicted an abnormal RNFL probability color code correlated with the VF report. Case B is a Caucasian glaucoma patient, where only the ML using compensated RNFL correctly predicted an abnormal RNFL probability color code, which agrees better with the VF report.

## Discussion

In this prospective duo-center, cross-sectional diagnostic study, we validated ML models for glaucoma detection, one trained using measured RNFL thickness and the other compensated RNFL thickness, derived from a multiple regression model fitted on healthy subjects that corrects the RNFL profile for factors like ethnicity, disc parameters and age. The results showed a poor reproducibility of the performance with the ML model trained on original RNFL data across different datasets. In contrast, the performance of the ML model trained on compensated RNFL seemed to be maintained. To the best of our knowledge, our study is the first to assess the performance of ML classifiers to detect glaucoma between ethnicities. Evaluating the performance of ML models for glaucoma detection using OCT data in new subjects is a crucial step in understanding the model generalizability to other populations.

Studies using ML approaches to detect glaucoma have used RNFL thickness data from OCT scans^5–9,38^. While most reported high cross-validated AUCs of 0.88–0.98 (internal validation)^5,6,8,9,38^, none assessed the model performance across ethnicities. In the current study, while the performance of ML when using measured RNFL thickness was excellent in the Asian dataset (AUC = 0.96), it was less promising in the Caucasian dataset (AUC = 0.83). Instead, the ML model trained on compensated RNFL thickness data (AUC = 0.93; P < 0.01) achieved better glaucoma detection than the traditional ML model (uses original RNFL data; AUC = 0.83) in an external dataset. The improvement in AUC is likely because the compensation model “normalizes” the RNFL profile (not biased by anatomic variability of each eye), which enhances the true extent of pathological damage for ML modeling. Most importantly, our study suggests that the anatomical variations may explain the poor generalizability of ML models when tested on datasets they were not trained on. It is well-known that there are considerable differences in the fundus between Asians and Caucasians. For instance, differences in optic disc size, shape, and cup-to-disc ratio have been reported across ethnicities/races in healthy individuals^14^. This is also evident from the current study where the ocular characteristics differed between training and external datasets. These anatomic differences can notoriously affect the RNFL thickness^15–21^. Hence, current ML models trained on measured RNFL data from a specific patient population may be less applicable across ethnicities. In general, the direct application of the ML model may be the most appropriate for individuals who share multiple characteristics from which the model was derived. In the future, it may be more feasible to use compensated RNFL thickness data to develop an ML classifier rather than using RNFL thickness data from specific ethnicity. Another reason for the heterogeneous model performance may be the distribution of glaucoma cases. The training Asian dataset had a more significant proportion of glaucoma than the testing Caucasian dataset (50 vs. 38%). Furthermore, the percentage of mild glaucoma in the testing Caucasian dataset was higher than in the training Asian dataset (75 vs. 61%). As a result, the higher percentage of mild glaucoma and normal eyes in the testing Caucasian dataset as compared to the training Asian dataset may make discrimination more difficult^39^. This can also be seen in the poor diagnostic performance of RNFL thickness in the testing Caucasian dataset compared to the testing Asian dataset.

The compensation model did not improve glaucoma detection compared to the conventional circumpapillary RNFL thickness in the Asian dataset, which was in contrast to our recent study^23^. One potential explanation is that the current study was a race-matched dataset, whereas our previous study^23^ was not matched for the race. Significant racial differences in the ocular factors were reported in a multi-ethnic Asian population^30^. Given that race is now matched equally between the training and test dataset, the influence of anatomical factors on the RNFL thickness is relatively minimal.

Our study had several limitations. First, the ground truth of glaucoma was defined clinically based on glaucomatous structural damage and corresponding VF abnormalities, which can be contentious even among expert observers^40^. Even though glaucoma diagnosis was made by board-certified ophthalmologists for both Asians and Caucasians, misclassifications as a result of this subjective assessment cannot be entirely ruled out, particularly in differentiating patients with early glaucoma. Second, our ML model was trained on Asians of Chinese, Malay, and Indian ethnicities and validated on Caucasians. The results reported herein apply to the sample and ML classifiers investigated and should be extrapolated to other populations or models with caution. In this study, we cannot investigate whether the different performances are still observed when the model was trained using a Caucasian training dataset. Glaucoma imaging was introduced recently in Caucasians, and recruitment is ongoing.

## Conclusion

In conclusion, although ML^5–9,38^ has been suggested as a potential solution for accurately and quickly identifying glaucomatous damage on diagnostic tests compared to subjective ocular examination and other traditional methods, their predictive performance can be variable across datasets.

## Supplementary Information

Supplementary Information.

## Data Availability

Data in this study cannot be shared publicly due to regulations of local ethical committees (Sing-Health Centralised Institutional Review Board, R1500/83/2017) and (Emergency University Hospital Bucharest Ethics Committee, 11285). Data might be made available to researchers who meet the criteria (to be provided once all data are available) for access to confidential data and upon Institutional Review Board's approval; requests for access to Asian dataset can be made to Prof. Leopold Schmetterer (leopold.schmetterer@seri.com.sg) and Caucasian dataset to Prof. Alina Cherecheanu (alina_cherecheanu@yahoo.com).
