# Supplementary material for: Assessing the external validity of machine learning-based detection of glaucoma
Source: Sci Rep. 2023 Jan 11;13:558. doi: 10.1038/s41598-023-27783-1 (PMC9834286; doi:10.1038/s41598-023-27783-1)
Supplement: Supplementary file 1 — Supplementary Information. [file 41598_2023_27783_MOESM1_ESM.docx]

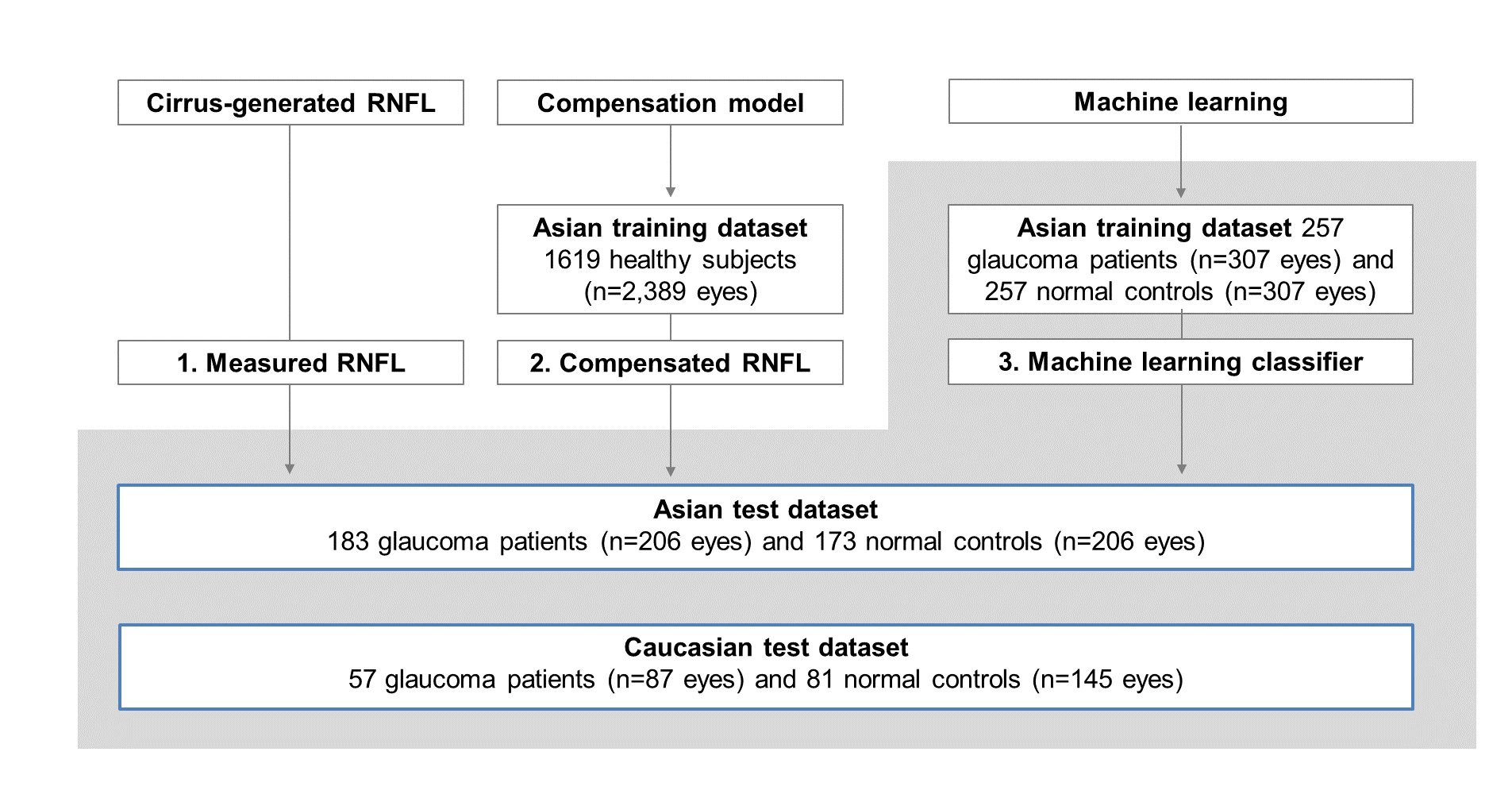


**Figure S1.** Flow diagram indicating the datasets used for training and testing the compensation model and machine learning approaches. Measured retinal nerve fiber layer (RNFL) thickness of the test datasets were included for comparison. The shaded region indicates the analyses performed for this study.


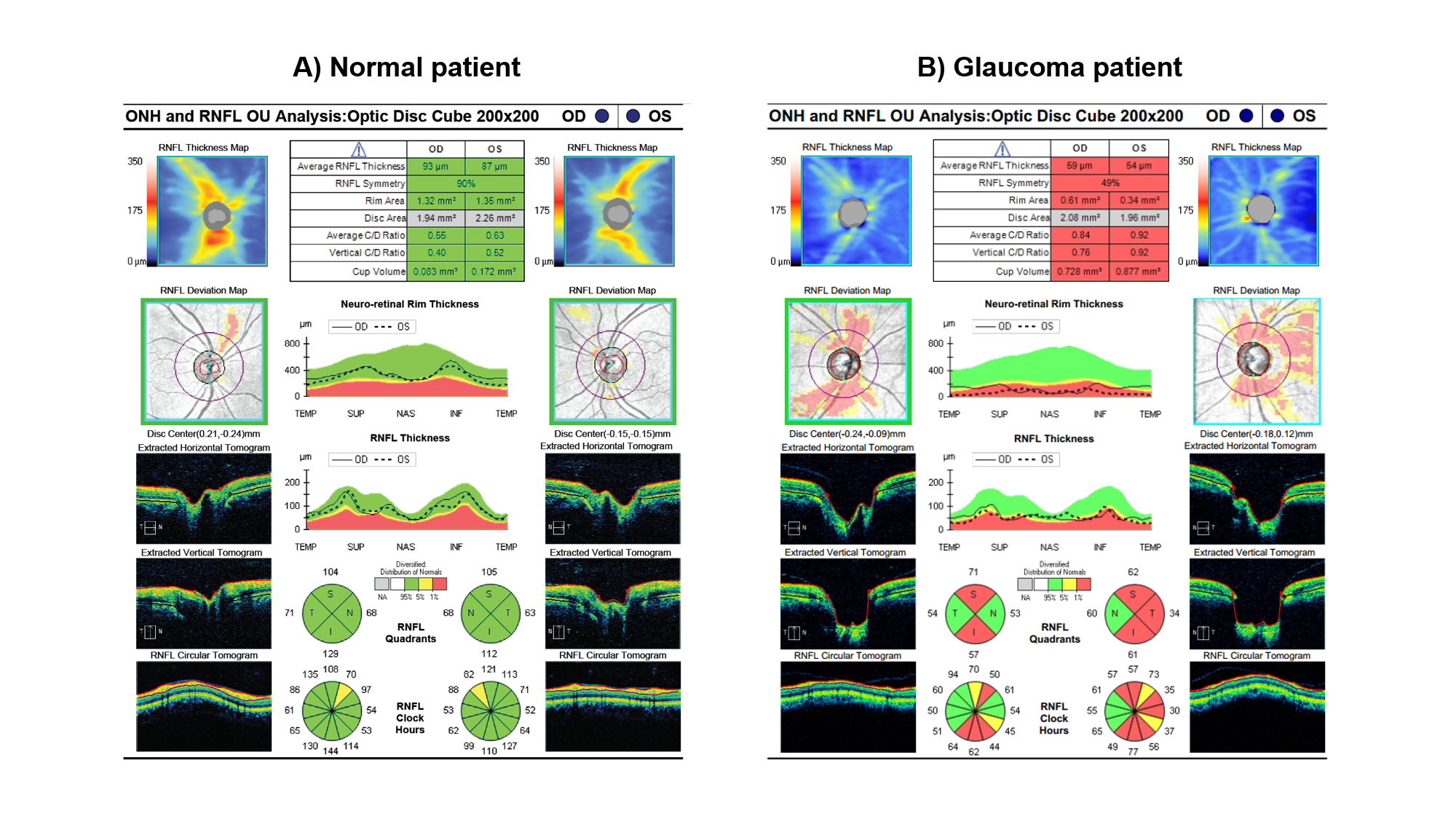


**Figure S2.** Two representative OCT images of A) normal and B) glaucoma patients.

| **Table S1. Comparison of glaucoma detection between measured retinal nerve fibre layer (RNFL), compensated RNFL, and machine learning approaches in the testing Caucasian balanced dataset** | | | | | |
| --- | --- | --- | --- | --- | --- |
|  | **Area under the receiver operating characteristic curve** | **Sensitivity at 95%** | **P value** | | |
| **Testing Caucasian balanced dataset** |  |  |  |  |  |
| 1. Measured RNFL | 0.82 (0.76-0.88) | 0.53 | Reference |  |  |
| 2. Compensated RNFL | 0.91 (0.86-0.95) | 0.64 | **<0.001** | Reference |  |
| 3. Machine learning (with measured RNFL) | 0.84 (0.78-0.89) | 0.53 | 0.271 | **<0.001** | Reference |
| 4. Machine learning (with compensated RNFL) | 0.92 (0.88-0.95) | 0.63 | **<0.001** | 0.529 | **<0.001** |
| Data in parentheses are 95% confidence intervals. | | | | | |
| Results for sensitivity are expressed as percentages. | | | | | |
| P value was obtained with DeLong et al. (1988) for paired receiver operating characteristic curves and values in bold indicate statistical significance. | | | | | |

| **Table S2. Performance matrix for testing dataset** | | | | | | |
| --- | --- | --- | --- | --- | --- | --- |
|  | **ML Models^** | **AUC** | **Sensitivity** | **Specificity** | **Accuracy** | **F1** |
| **Testing Caucasian dataset** | | | | | | |
| (a) Machine learning (with measured RNFL) | LR | 0.964 | 0.864 | 0.786 | 0.876 | 0.883 |
|  | SVM | 0.952 | 0.825 | 0.723 | 0.862 | 0.871 |
|  | RF | 0.965 | 0.854 | 0.772 | 0.874 | 0.879 |
|  | GB | 0.961 | 0.823 | 0.825 | 0.903 | 0.907 |
| (b) Machine learning (with compensated RNFL) | LR | 0.959 | 0.825 | 0.748 | 0.871 | 0.878 |
|  | SVM | 0.95 | 0.796 | 0.709 | 0.859 | 0.868 |
|  | RF | 0.96 | 0.823 | 0.762 | 0.876 | 0.881 |
|  | GB | 0.954 | 0.833 | 0.748 | 0.896 | 0.897 |
| **Testing Caucasian dataset** | | | | | | |
| (c) Machine learning (with measured RNFL) | LR | 0.808 | 0.506 | 0.186 | 0.793 | 0.696 |
|  | SVM | 0.792 | 0.517 | 0.152 | 0.78 | 0.653 |
|  | RF | 0.843 | 0.494 | 0.379 | 0.776 | 0.671 |
|  | GB | 0.853 | 0.517 | 0.386 | 0.785 | 0.68 |
| (d) Machine learning (with compensated RNFL) | LR | 0.926 | 0.701 | 0.69 | 0.853 | 0.776 |
|  | SVM | 0.92 | 0.701 | 0.628 | 0.828 | 0.726 |
|  | RF | 0.917 | 0.667 | 0.628 | 0.841 | 0.752 |
|  | GB | 0.903 | 0.655 | 0.552 | 0.832 | 0.735 |
|  |  |  |  |  |  |  |

^ML = Machine Learning; LR = Logistic Regression; SVM = Support Vector Machines; RF = Random Forests; GB = Gradient Boosting

| **Table S3. Confusion matrix for testing dataset** | | | | | |  |  |  |  |
| --- | --- | --- | --- | --- | --- | --- | --- | --- | --- |
| **Predicted Values\ Actual Values** | | **LR** |  | **SVM** |  | **RF** |  | **GB** |  |
|  |  | **Normal** | **Glaucoma** | **Normal** | **Glaucoma** | **Normal** | **Glaucoma** | **Normal** | **Glaucoma** |
| **Testing Asian dataset** | | | | | | | | | |
| (a) Machine learning (with measured RNFL) | Normal | 169 | 14 | 163 | 14 | 171 | 17 | 178 | 12 |
|  | Glaucoma | 37 | 192 | 43 | 192 | 35 | 189 | 28 | 194 |
| (b) Machine learning (with compensated RNFL) | Normal | 168 | 15 | 163 | 15 | 172 | 17 | 181 | 18 |
|  | Glaucoma | 38 | 191 | 43 | 191 | 34 | 189 | 25 | 188 |
| **Testing Caucasian dataset** | | | | | | | | | |
| (c) Machine learning (with measured RNFL) | Normal | 129 | 32 | 133 | 39 | 127 | 34 | 129 | 34 |
|  | Glaucoma | 16 | 55 | 12 | 48 | 18 | 53 | 16 | 53 |
| (d) Machine learning (with compensated RNFL) | Normal | 139 | 28 | 139 | 34 | 139 | 31 | 139 | 33 |
|  | Glaucoma | 6 | 59 | 6 | 53 | 6 | 56 | 6 | 54 |

LR = Logistic Regression; SVM = Support Vector Machines; RF = Random Forests; GB = Gradient Boosting
